# Exercise Heat Acclimation With Dehydration Does Not Affect Vascular and Cardiac Volumes or Systemic Hemodynamics During Endurance Exercise

**DOI:** 10.3389/fphys.2021.740121

**Published:** 2021-11-12

**Authors:** Gavin Travers, José González-Alonso, Nathan Riding, David Nichols, Anthony Shaw, Julien D. Périard

**Affiliations:** ^1^Athlete Health and Performance Research Centre, Aspetar Orthopaedic and Sports Medicine Hospital, Doha, Qatar; ^2^Centre for Human Performance and Rehabilitation, College of Health, Medicine and Life Sciences, Brunel University London, Uxbridge, United Kingdom; ^3^Division of Sport, Health and Exercise Sciences, Department of Life Sciences, College of Health, Medicine and Life Sciences, Brunel University London, Uxbridge, United Kingdom; ^4^Sport Development Centre, Loughborough University, Loughborough, United Kingdom; ^5^Research Institute for Sport and Exercise, University of Canberra, Bruce, ACT, Australia

**Keywords:** heat acclimatization, blood volume, plasma volume, stroke volume, heat stress

## Abstract

Permissive dehydration during exercise heat acclimation (HA) may enhance hematological and cardiovascular adaptations and thus acute responses to prolonged exercise. However, the independent role of permissive dehydration on vascular and cardiac volumes, ventricular-arterial (VA) coupling and systemic hemodynamics has not been systematically investigated. Seven males completed two 10-day exercise HA interventions with controlled heart rate (HR) where euhydration was maintained or permissive dehydration (-2.9 ± 0.5% body mass) occurred. Two experimental trials were conducted before and after each HA intervention where euhydration was maintained (-0.5 ± 0.4%) or dehydration was induced (-3.6 ± 0.6%) *via* prescribed fluid intakes. Rectal (T_re_) and skin temperatures, HR, blood (BV) and left ventricular (LV) volumes, and systemic hemodynamics were measured at rest and during bouts of semi-recumbent cycling (55% V̇O_2__peak_) in 33°C at 20, 100, and 180 min. Throughout HA sweat rate (12 ± 9%) and power output (18 ± 7 W) increased (*P* < 0.05), whereas T_re_ was 38.4 ± 0.2°C during the 75 min of HR controlled exercise (*P* = 1.00). Neither HA intervention altered resting and euhydrated exercising T_re_, BV, LV diastolic and systolic volumes, systemic hemodynamics, and VA coupling (*P* > 0.05). Furthermore, the thermal and cardiovascular strain during exercise with acute dehydration post-HA was not influenced by HA hydration strategy. Instead, elevations in T_re_ and HR and reductions in BV and cardiac output matched pre-HA levels (*P* > 0.05). These findings indicate that permissive dehydration during exercise HA with controlled HR and maintained thermal stimulus does not affect hematological or cardiovascular responses during acute endurance exercise under moderate heat stress with maintained euhydration or moderate dehydration.

## Introduction

Heat acclimation (HA) increases the rate and sensitivity of sweating and skin blood flow, and attenuates the rise in whole body temperature and heart rate (HR) during submaximal exercise in the heat (Fox et al., 1963; Rowell et al., 1967; Nadel et al., 1974; Roberts et al., 1977). Adaptations to HA vary and depend on the type of intervention (i.e., active or passive), participant fitness status, environmental conditions (i.e., dry or humid heat), and possibly the hydration strategy adopted (i.e., maintained euhydration or dehydration *via* fluid restriction) (Taylor, 2014; Périard et al., 2015). Typically, HA results in an expansion in plasma volume (PV), which has historically been considered a transient response to euhydrated exercise interventions; initially increasing after 5–7 exposures before decreasing slightly thereafter (Wyndham et al., 1968; Taylor, 2014; Périard et al., 2015). Together with a decreased HR, a greater PV and therefore total blood volume (BV) is proposed to support the diastolic filling of the left ventricle (LV) (Wyndham et al., 1968; Senay, 1986), such that stroke volume (SV) may be increased during exercise in the heat (Rowell et al., 1967; Wyndham et al., 1968; Nielsen et al., 1993). Recently, however, our own work and that of others has demonstrated that both passive HA (Trachsel et al., 2020) and exercise HA with maintained euhydration (Travers et al., 2020a) had minimal effects on LV volumes, function and systemic hemodynamics when PV and HR were similar pre- to post-HA.

Conversely, acute permissive dehydration beyond 2% of body mass *via* fluid restriction results in hyperosmotic hypovolemia, promoting renal water conservation following the release of arginine vasopressin and aldosterone (Nagashima et al., 2001; Kenefick et al., 2007). Furthermore, permissive dehydration *via* fluid restriction during controlled hyperthermia HA has been demonstrated to result in greater attenuations in exercising HR, increases in post-exercise aldosterone, and elevations in resting PV when nine trained individuals restricted fluid during a 5-day intervention, compared to when the same individuals maintained pre-exercise euhydration daily (Garrett et al., 2014). This hypervolemic state can purportedly be maintained throughout protocols lasting up to 28 days (Patterson et al., 2004b, 2014) when fluid regulatory responses are consistently stimulated *via* dehydration and a constant thermal impulse for adaptation is provided (i.e., controlled hyperthermia HA) (Taylor, 2014). However, the intra-individual responses to HA with altered fluid intake strategies is limited to a small sample of fit, healthy individuals that demonstrate varying responses within the literature. For example, while some have demonstrated greater relative expansion of PV and lowered exercising HR following HA with dehydration compared to maintaining euhydration (Garrett et al., 2014), this has not been observed by others (Neal et al., 2016b). Whether restricting fluid intake during exercise HA provides a greater stimulus for hematological and cardiovascular adaptations that enhance vascular, cardiac, or hemodynamic stability during exercise in the heat remains unclear.

As we have demonstrated previously, LV diastolic or systolic function at rest and during exercise-induced dehydration >3% were not altered after 10 days of euhydrated exercise HA (Travers et al., 2020a). Instead, LV diastolic filling was impaired alongside similar reductions in BV and elevations in HR both pre- and post-HA. Moreover, it has also been demonstrated that despite a significant expansion in PV and BV following controlled hyperthermia HA with fluid restriction, acute exercise-induced dehydration (∼2–3%) results in larger relative reductions in BV, while core temperature and HR are lowered (Patterson et al., 2004a, 2014). When undertaking exercise in the heat in a hypohydrated state (i.e., 3–5% body mass deficit *via* fluid restriction or diuretic administration), improved or similar core temperature, HR and BV responses have been observed following HA (Buskirk et al., 1958; Sawka et al., 1983; Cheung and Mclellan, 1998; Ikegawa et al., 2011). Together, these findings suggest that both HA hydration strategy and HA status may independently influence vascular and cardiac volumes during acute exercise-induced dehydration. However, whether fluid restriction during HA blunts impairments in diastolic filling compared to a euhydrated HA intervention during acute exercise-induced dehydration is yet to be determined.

The purpose of this study was to determine whether daily dehydration during exercise with a controlled HR influences the BV and HR responses to HA. A second aim was to determine whether fluid intake strategy during HA alters vascular and cardiac volumes and central hemodynamics during exercise in the heat with and without acute dehydration. Responses to 10 days of HA with matched levels of dehydration were compared to HA with maintained euhydration in the same individuals. It was hypothesized that dehydrated HA would further increase BV and lower HR compared to euhydrated HA, improving the diastolic filling of the LV, and thus enhance SV during exercise under heat stress with both maintained euhydration and mild exercise-induced dehydration (<2% body mass loss). However, pronounced dehydration (>3% body mass loss), regardless of HA fluid intake strategy, would result in lower exercising Q̇ compared to euhydration, associated in part with elevated body temperatures and HR and reductions in BV and diastolic filling of the LV.

## Materials and Methods

### Participants

**FIGURE 1:**
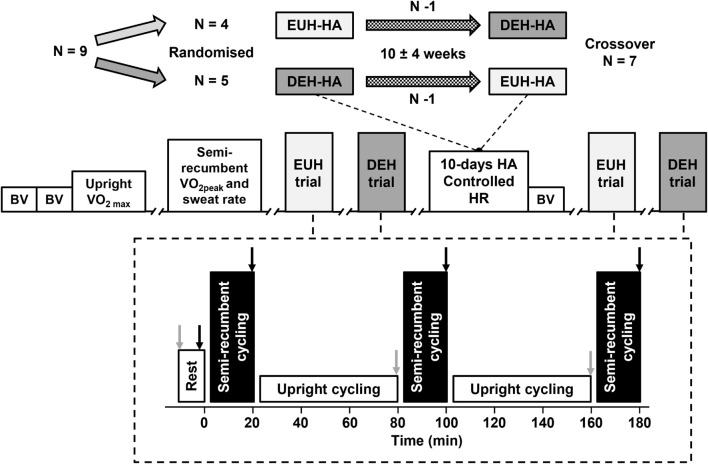
**Top:** Schematic of randomized crossover trial design for seven participants following one withdrawal after each initial heat acclimation (HA) intervention. Each intervention consisted of 10 days of HA with controlled heart rate (HR) and was separated by a 10 ± 4 week washout period. Truncations indicate minimum of 24 h between trials. **Bottom:** euhydration (EUH) and dehydration (DEH) experimental trial design. Experimental trials order was randomized between participants and repeated in the second intervention. Gray arrows: measurements of nude body mass. Black arrows: measurements of body temperature, blood volume, central hemodynamics, and left ventricular volumes. BV: carbon monoxide rebreathing for the measurement of blood volume. V̇O_2__max_: maximal oxygen uptake during upright cycling exercise. V̇O_2__peak_ and sweat rate: peak oxygen uptake during semi-recumbent cycling and pre-HA sweat rate estimation.

A total of nine male participants were recruited to complete two HA interventions in a randomized crossover design study. However, two participants withdrew prior to completion of the second arm of the study due to unforeseen circumstances. Therefore, data are presented for the seven participants who completed both HA interventions (Figure 1). Data from the participants that completed the euhydrated HA intervention have been published as part of a larger cohort (Travers et al., 2020a). Participants were all trained cyclists and triathletes regularly undertaking >5 h of endurance training per week and were non-native residents of Qatar. Their characteristics on enrollment to the study are presented in Table 1. All participants underwent a pre-screening procedure consisting of a health questionnaire and echocardiographic assessment prior to providing written informed consent. The study was approved by Anti-Doping Lab Qatar Research Ethics Committee (Approval no. F201500105) and conducted in accordance with the Declaration of Helsinki.

**TABLE 1:** Participant characteristics and summary of experimental workloads in the euhydrated (EUH-HA) and dehydrated (DEH-HA) heat acclimation (HA) interventions.

	EUH-HA	DEH-HA
Age (years)	34 ± 4	
Height (cm)	175 ± 5	
Body mass (kg)	75.2 ± 5.1	75.4 ± 4.9
V̇O_2__max_ (mL kg min^–1^)	52 ± 07	53 ± 7
Upright cycling workload (W)	170 ± 22	171 ± 25
Semi-recumbent cycling workload (W)	133 ± 18	136 ± 17
HA target heart rate (beats min^–1^)	146 ± 7	144 ± 7
Daily change in pre-exercise body mass (%)	-0.6 ± 0.3	-2.9 ± 0.5*

Data are mean ± SD for n = 7. *Significantly different from EUH-HA, P < 0.001.

### Experimental Design

Two exercise-HA interventions were separated by an average washout period of 10 ± 4 weeks (Figure 1). Each period of HA consisted of 10 days of exercise at a controlled HR in 40°C and 40% relative humidity (RH) for 90 min per day. Fluid intake was prescribed to either maintain pre-exercise euhydration or elicit similar levels of exercise-induced dehydration and was altered daily to match any changes noted in sweating rate with HA. The initial 15 min workload was equivalent to 65% of maximal oxygen uptake (V̇O_2__max_), thereafter workload was adjusted to maintain a HR corresponding to that intensity (Travers et al., 2020b; Table 1). Prior to and following each HA intervention, two experimental trials were conducted in a randomized order. The experimental trials determined the hematological, hemodynamic, and LV volume responses to prolonged exercise in 33.0 ± 0.3°C and 50 ± 4% RH while euhydrated or with matched levels of dehydration. Trial order was maintained following each HA period and replicated in the subsequent intervention. Participants were encouraged to minimize outdoor exercise exposure for a minimum of 3 weeks prior to each arm of the study, and this was a minimum requirement for the two participants completing the study between May and August. These two participants reported regular indoor exercise (i.e., cycle ergometer/treadmill running in air conditioning) and long-term (4–6 weeks) travel from the region outside of the study.

### Pre-experimental Procedures

Participants attended the laboratory on four occasions prior to the first experimental trial (Figure 1). During two visits separated by a minimum of 24 h, maximal and peak incremental tests to volitional exhaustion were conducted during upright (Lode Excalibur Sport, Groningen, Netherlands) and semi-recumbent (Ergoselect, Ergoline, GMbH, Germany) cycling exercise, respectively. Each test was used to determine the workloads and target HR for the experimental trials and HA sessions. Tests were conducted in a cool environment (19.2 ± 1.9°C and 63 ± 10% RH) and participants wore cycling shorts, socks and shoes throughout. Following completion of the semi-recumbent test, participants rested in the laboratory for 30 min to allow for body temperature to return to pre-exercise levels. Thereafter, they entered an environmental chamber (TEMI 1000, Sanwood Environmental Chambers co., Taiwan) with target ambient conditions of 33°C and 50% RH and completed 60 min of upright cycling exercise at a workload equivalent to 65% V̇O_2__max_. *Ad libitum* fluid intake was recorded and used to correct for changes in nude body mass for the calculation of hourly whole-body sweat rate pre-HA. Hemoglobin mass was determined in duplicate on consecutive days *via* carbon monoxide rebreathing (Schmidt and Prommer, 2005) prior to each HA intervention. This test was repeated once following HA to determine the effects of each intervention on hemoglobin mass. The typical error of this procedure was 0.63%.

### Experimental Trials

Experimental procedures were identical to those used previously (Travers et al., 2020a). Briefly, following confirmation of euhydration *via* measurements of urine specific gravity (Armstrong et al., 1994), nude body mass was determined to the nearest 100 g (SECA 798, Germany). Participants then self-inserted a rectal thermistor (DM 852, Ellab, A/S, Hillerød, Denmark) and donned a HR monitor (RS800CX, T31-Coded Transmitter, Polar Electro, Kempele, Finland), cycling shorts, socks and shoes before lying supine in the main laboratory. A cannula was inserted into a right antecubital vein and flushed with 2 mL of saline before participants were instrumented with electrocardiogram sensors and skin temperature thermistors (iButtons, Maxim Integrated Products, Sunnyvale, CA, United States). Following a 10 min period of rest, HR and duplicate measurements of blood pressure were recorded. A resting blood sample was then collected before participants entered an environmental chamber and mounted a semi-recumbent cycle ergometer. The ergometer was tilted longitudinally and resting echocardiographic images were recorded following 5 min of rest. Participants then cycled in a semi-recumbent position at a workload equivalent to 55% V̇O_2__peak_ (135 ± 18 W) for 5 min. Blood pressure was then recorded and the ergometer was again tilted for measurements of exercising LV volumes, mean arterial pressure (MAP) and blood volume over a further 15 min.

Following the initial resting and exercising measurements on the semi-recumbent cycle ergometer, participants transferred to the upright cycle ergometer and exercised for 60 min at 65% V̇O_2__max_ (171 ± 23 W). Fluid intake was prescribed over this period and consisted of a 0.1% electrolyte drink (HIGH5 ZERO, H5, Bardon, United Kingdom) divided into four equal aliquots to the nearest 1 mL. The total volume consumed was equivalent to either 90% (euhydration; EUH) or 10% (dehydration; DEH) of expected hourly sweat loss and was calculated from the pre-experimental visit and the final day of HA for the pre- and post-HA trials, respectively. Intake began at the onset of upright exercise and subsequent aliquots were provided after 15, 30, and 45 min of cycling. A fan directed at participants throughout all periods of exercise provided 3 m s^–1^ convective airflow. At the end of each period of upright exercise, participants removed all clothing except instrumentation and towel dried non-evaporated sweat before body mass was measured within the chamber to determine changes in total body water. Participants then re-dressed, mounted the semi-recumbent ergometer and completed the exercising echocardiographic measurement period. This process was repeated a final time before the end of the trial, following another 60 min of upright cycling exercise. Altogether, measurements of MAP, BV, and LV volumes and function were conducted at rest and after 20, 100, and 180 min of dynamic exercise in the heat (Figure 1).

### Measurement Procedures

Rectal temperature (T_re_) was measured *via* an individualized re-usable thermistor placed 15 cm beyond the anal sphincter. Area weighted mean skin temperature (T_sk_) was calculated from four sites using the equation of Ramanathan (1964). T_re_ and T_sk_ were recorded at rest and at the beginning and end of each semi-recumbent exercise period. HR and T_re_ were also recorded every 5 min during upright cycling, whereas ratings of perceived exertion (Borg, 1982) and thermal comfort (Bedford, 1936) were recorded every 10 min. Venous blood samples (2 mL) were drawn into lithium heparinized syringes (PICO 50, Radiometer) following a ∼2 mL discharge immediately after echocardiographic measurements. The cannula was then flushed with ∼5 mL of saline. Whole blood was immediately analyzed in triplicate for measurements of hemoglobin concentration and hematocrit (ABL90 FLEX, Radiometer, BrØnshØj, Denmark). Absolute BV, PV, and red cell volume were calculated as outlined previously (Travers et al., 2020a).

Echocardiographic images were recorded by a single sonographer at a standardized order and depth for each participant. All images were collected using a commercially available ultrasound machine (CX50 POC, Philips Healthcare, Netherlands) and 5 MHz sector array probe (S5-1, Philips Healthcare). Frame rate was fixed at 60 Hz for 2D image acquisition. A minimum of six cardiac cycles were recorded of each view and analysis was conducted over three consecutive cardiac cycles where possible. All images were recorded at the end of expiration. Apical 2- and 4-chamber images were acquired at all time points. Recordings of the short-axis base were also made at rest to determine the effect of exercise HA on LV mass (Schiller et al., 1989). All images were exported, de-identified and analyzed offline (Q Station 3.0, Philips Healthcare, Netherlands) at the end of the data collection period as to avoid confirmation bias. Diastolic and systolic LV volumes were determined using the Simpson’s method of bi-plane disk summation while HR was recorded *via* a 3-lead electrocardiogram inherent in the ultrasound. The co-efficient of variation for repeated exercising hemodynamics using echocardiography was between 6 and 10%. Q̇ was calculated as the product of HR and SV. MAP was measured manually in duplicate using a sphygmomanometer and calculated as [(2 × DBP) + SBP]/3, where DBP and SBP are diastolic and systolic blood pressures, respectively. Systemic vascular resistance (SVR) was calculated as MAP/Q̇. Effective arterial elastance was calculated as 0.9 × SBP/SV. LV end-systolic elastance was calculated as 0.9 × SBP/ESV, where ESV is end-systolic volume (Chantler et al., 2008). Ventricular-arterial (VA) coupling was calculated as the quotient of end-systolic elastance and effective arterial elastance (Takeuchi et al., 1991).

### Statistical Analyses

An *a priori* sample size estimation was conducted using G^∗^Power (version 3.1.9.6) based on observed changes in SV and Q̇ reported in a recent meta-analysis (Tyler et al., 2016). With an alpha level of 0.05, required power (1-β) of 0.8 and correlation of repeated measures of 0.5, a within-factors ANOVA with 2 groups (HA interventions) and 16 repeated measures (4 experimental trials, each with 4 measurement points) would require a minimum of 6 participants. Three-way ANOVA (trial × HA time × HA intervention) with repeated measures analyses were used to compare measurements at rest. Separate three-way ANOVA’s for effects of prolonged exercise (HA intervention × trial × time) and average exercising responses following HA (HA intervention × HA time × hydration) were used to compare measurements during semi-recumbent cycling. Mauchley’s test was used to test the assumption of Sphericity. In cases where this assumption was violated a Greenhouse-Geisser correction factor was applied. Bonferroni *post hoc* testing was employed to determine where differences occurred. All statistical analyses were conducted using SPSS (Version 21, IBM, Armonk, United States). The level of significance was set at *P* < 0.05.

## Results

### Responses Throughout the Heat Acclimation Interventions

There were no main effects of hydration strategy (i.e., euhydrated HA *versus* HA with daily dehydration) on thermal or cardiovascular HA adaptations during the 10-day interventions (all *P* > 0.05). Both interventions resulted in a 12 ± 9% (0.2 ± 0.1 L h^–1^) increase in hourly sweat rate (*P* = 0.016) and 0.5 ± 0.4°C reduction in exercising T_sk_ (*P* = 0.007). Daily changes in body mass following exercise were similar between days of HA within each intervention (*P* = 0.083, Table 1). T_re_ after the initial 15 min period of fixed workload exercise was not altered between days 1 and 10 of HA in either condition (*P* = 1.00). However, 15 min HR was 9 ± 5 beats min^–1^ lower on day 10 of HA in both interventions (*P* < 0.002). The workload necessary to maintain the prescribed HR for the final 75 min of exercise increased with HA (*P* = 0.001). The increase in workload between days 1 and 10 of HA was greater with euhydrated HA (26 ± 11 W) than dehydrated HA (11 ± 12 W, *P* = 0.008).

### Effect of Heat Acclimation on Resting Responses

Participants attended the pre- and post-HA experimental trials in a well hydrated state as indicated by similar USG values (1.014 ± 0.007, *P* = 0.187). Resting thermal, hematological and hemodynamic responses following euhydrated and dehydrated HA are presented in Tables 2, 3, respectively. There were no main effects of HA on resting T_re_ (*P* = 0.532), PV (*P* = 0.090), red cell volume (2686 ± 201 mL, *P* = 0.198), or total BV (*P* = 0.177), which were similar between HA interventions (all *P* > 0.05). HA had no effect on LV mass (*P* = 0.183), averaging 173 ± 9 g pre- and 173 ± 9 g post-HA, respectively. There was a main effect of HA on resting SV (*P* = 0.022) but no interaction effects (all *P* > 0.05). The small (5 ± 7 mL) increase in SV following HA was not associated with a discernible change in EDV (*P* = 0.058) or ESV (*P* = 0.754). Furthermore, neither resting Q̇ (5.31 ± 0.72 L min^–1^ pre-HA and 5.49 ± 0.99 L min^–1^ post-HA; *P* = 0.226), nor resting MAP or SVR were affected by HA (*P* = 0.478 and 0.330, respectively; Tables 2, 3).

**TABLE 2:** Thermal, hematological, and hemodynamic responses at rest and at 20, 100, and 180 min of semi-recumbent cycling in moderate heat with maintained euhydration (EUH) or progressive exercise-induced dehydration (DEH), before (pre) and after (post) euhydrated heat acclimation (HA) with controlled heart rate.

			Time (min)			
	Trial	HA	Rest	20 min	100 min	180 min
T_re_ (°C)	EUH	Pre	37.1 ± 0.3	37.4 ± 0.3	38.2 ± 0.4	38.4 ± 0.2
		Post	37.0 ± 0.3	37.3 ± 0.2	38.1 ± 0.1	38.3 ± 0.1
	DEH	Pre	36.8 ± 0.4	37.3 ± 0.3	38.4 ± 0.3^‡^	38.9 ± 0.3^‡^
		Post	36.9 ± 0.3	37.2 ± 0.3	38.3 ± 0.3	38.9 ± 0.5^‡^
T_sk_ (°C)	EUH	Pre	33.6 ± 0.7	34.1 ± 0.5	34.1 ± 1.0	34.1 ± 1.3
		Post	33.5 ± 1.0	34.0 ± 0.6	33.8 ± 0.9	34.0 ± 1.0
	DEH	Pre	33.5 ± 0.7	33.9 ± 0.7	34.3 ± 0.6	34.0 ± 0.9
		Post	33.4 ± 0.8	34.0 ± 0.6	33.8 ± 0.9	34.0 ± 0.8
BV (mL)	EUH	Pre	5996 ± 486	5638 ± 480	5654 ± 463	5653 ± 430
		Post	6048 ± 450	5732 ± 433	5752 ± 454	5747 ± 415
	DEH	Pre	6003 ± 505	5636 ± 491	5488 ± 419	5253 ± 404^‡^
		Post	6117 ± 400	5737 ± 387	5593 ± 325	5367 ± 311^‡^
Q̇ (L min^–1^)	EUH	Pre	5.5 ± 0.7	13.3 ± 1.6	13.6 ± 1.5	14.1 ± 1.7
		Post	5.2 ± 0.6	13.5 ± 1.4	14.5 ± 1.6	14.8 ± 1.5
	DEH	Pre	5.3 ± 0.6	13.7 ± 1.9	13.4 ± 1.8	12.5 ± 1.7^‡^
		Post	5.5 ± 1.0	13.5 ± 1.5	14.2 ± 1.4	12.7 ± 1.4^‡^
HR (beats min^–1^)	EUH	Pre	62 ± 7	121 ± 7	125 ± 7	138 ± 6
		Post	54 ± 4*	119 ± 5	127 ± 7	132 ± 4
	DEH	Pre	60 ± 7	122 ± 6	137 ± 9^‡^	149 ± 10^‡^
		Post	57 ± 5	119 ± 3	132 ± 8	149 ± 9^‡^
SV (mL)	EUH	Pre	89 ± 11	110 ± 11	109 ± 17	102 ± 9
		Post	95 ± 13*	113 ± 12	114 ± 15	112 ± 12
	DEH	Pre	89 ± 6	112 ± 13	99 ± 13^‡^	84 ± 9^‡^
		Post	96 ± 10*	113 ± 11	107 ± 10	85 ± 8^‡^
EDV (mL)	EUH	Pre	138 ± 20	150 ± 19	152 ± 24	143 ± 19
		Post	146 ± 21	151 ± 26	155 ± 24	152 ± 23
	DEH	Pre	138 ± 12	151 ± 20	136 ± 20^‡^	121 ± 20^‡^
		Post	144 ± 20	152 ± 17	140 ± 19^‡^	121 ± 11^‡^
ESV (mL)	EUH	Pre	49 ± 10	40 ± 10	43 ± 10	41 ± 12
		Post	51 ± 11	38 ± 15	41 ± 10	40 ± 11
	DEH	Pre	49 ± 7	39 ± 10	38 ± 10	37 ± 13
		Post	49 ± 11	40 ± 10	33 ± 11	36 ± 5
MAP (mmHg)	EUH	Pre	83 ± 15	98 ± 13	94 ± 11	92 ± 10
		Post	80 ± 12	94 ± 9	92 ± 8	90 ± 6
	DEH	Pre	83 ± 13	94 ± 12	91 ± 16	86 ± 16
		Post	80 ± 5	93 ± 13	88 ± 8	83 ± 14
SVR (mmHg L min^–1^)	EUH	Pre	15.2 ± 1.7	7.4 ± 1.0	7.0 ± 0.7	6.6 ± 0.8
		Post	15.6 ± 2.0	7.0 ± 0.8	6.4 ± 0.8	6.1 ± 0.7
	DEH	Pre	15.7 ± 1.7	7.0 ± 1.0	6.8 ± 1.1	6.9 ± 1.1
		Post	14.8 ± 1.9	6.9 ± 1.0	6.3 ± 0.7	6.6 ± 1.2

Data are mean ± SD for n = 7. *P < 0.05 vs. pre-HA. ^‡^P < 0.05 vs. EUH trial. T_re_, Rectal temperature; T_sk_, Skin temperature; BV, Blood volume; Q̇, Cardiac output; HR, Heart rate; SV, Stroke volume; EDV, End-diastolic volume; ESV, End-systolic volume; MAP, Mean arterial pressure; SVR, Systemic vascular resistance.

**TABLE 3:** Thermal, hematological, and hemodynamic responses at rest and at 20, 100, and 180 min of semi-recumbent cycling in moderate heat with maintained euhydration (EUH) or progressive exercise-induced dehydration (DEH), before (pre) and after (post) dehydrated heat acclimation (HA) with controlled heart rate.

			Time (min)			
	Trial	HA	Rest	20 min	100 min	180 min
T_re_ (°C)	EUH	Pre	37.0 ± 0.3	37.4 ± 0.2	38.1 ± 0.3	38.4 ± 0.4
		Post	36.9 ± 0.2	37.3 ± 0.2	38.0 ± 0.3	38.2 ± 0.3
	DEH	Pre	36.9 ± 0.2	37.3 ± 0.3	38.4 ± 0.2^‡^	38.9 ± 0.4^‡^
		Post	36.8 ± 0.3	37.1 ± 0.3	38.3 ± 0.3	38.7 ± 0.3^‡^
T_*sk*_ (°C)	EUH	Pre	33.6 ± 0.4	34.0 ± 0.7	34.2 ± 1.0	34.3 ± 0.7
		Post	33.5 ± 0.6	34.2 ± 0.7	33.8 ± 0.8	34.1 ± 0.9
	DEH	Pre	33.7 ± 0.6	34.3 ± 0.6	34.3 ± 0.8	34.1 ± 1.1
		Post	33.2 ± 1.1	34.3 ± 0.6	34.3 ± 0.6	34.1 ± 0.8
BV (mL)	EUH	Pre	5996 ± 486	5713 ± 541	5766 ± 464	5750 ± 411
		Post	6048 ± 450	5715 ± 432	5761 ± 388	5760 ± 408
	DEH	Pre	6003 ± 505	5714 ± 450	5541 ± 374	5374 ± 391^‡^
		Post	6117 ± 400	5775 ± 486	5644 ± 448	5453 ± 400^‡^
Q̇ (L min^–1^)	EUH	Pre	5.2 ± 0.8	13.3 ± 2.3	13.8 ± 2.4	14.5 ± 2.5
		Post	5.7 ± 1.2	13.6 ± 1.9	13.7 ± 1.9	14.4 ± 2.0
	DEH	Pre	5.3 ± 0.9	13.6 ± 2.0	13.7 ± 2.4	12.3 ± 2.5^‡^
		Post	5.5 ± 1.2	13.8 ± 2.2	14.1 ± 2.0	12.5 ± 1.9^‡^
HR (beats min^–1^)	EUH	Pre	57 ± 8	122 ± 13	124 ± 9	133 ± 13
		Post	60 ± 10^†^	120 ± 8	125 ± 8	134 ± 9
	DEH	Pre	58 ± 5	121 ± 7	134 ± 11^‡^	145 ± 16^‡^
		Post	59 ± 8	123 ± 12	136 ± 9^‡^	144 ± 10^‡^
SV (mL)	EUH	Pre	91 ± 12	109 ± 13	111 ± 13	109 ± 12
		Post	95 ± 9*	113 ± 11	110 ± 12	108 ± 13
	DEH	Pre	91 ± 11	112 ± 13	101 ± 10^‡^	84 ± 10^‡^
		Post	93 ± 12*	112 ± 13	104 ± 11^‡^	86 ± 9^‡^
EDV (mL)	EUH	Pre	146 ± 20	155 ± 26	155 ± 24	155 ± 24
		Post	149 ± 17	159 ± 22	155 ± 21	154 ± 24
	DEH	Pre	147 ± 17	156 ± 19	145 ± 18^‡^	127 ± 18^‡^
		Post	148 ± 21	157 ± 20	151 ± 18	133 ± 18^‡^
ESV (mL)	EUH	Pre	55 ± 10	46 ± 17	45 ± 13	46 ± 12
		Post	54 ± 10	46 ± 13	45 ± 12	46 ± 12
	DEH	Pre	56 ± 10	44 ± 11	43 ± 10	43 ± 9
		Post	55 ± 10	45 ± 13	47 ± 13	46 ± 13
MAP (mmHg)	EUH	Pre	81 ± 12	90 ± 11	88 ± 11	87 ± 9
		Post	81 ± 12	92 ± 14	91 ± 11	88 ± 10
	DEH	Pre	82 ± 12	92 ± 16	88 ± 13	84 ± 10
		Post	84 ± 16	92 ± 17	90 ± 16	89 ± 14
SVR (mmHg L min^–1^)	EUH	Pre	15.6 ± 0.9	6.9 ± 0.9	6.5 ± 0.9	6.1 ± 0.8
		Post	14.4 ± 1.4	6.8 ± 0.8	6.6 ± 0.8	6.2 ± 0.8
	DEH	Pre	15.9 ± 2.1	6.8 ± 1.3	6.6 ± 1.3	7.0 ± 1.2
		Post	15.4 ± 2.0	6.7 ± 1.2	6.4 ± 1.0	7.3 ± 1.3

Data are mean ± SD for n = 7. *P < 0.05 vs. pre-HA. ^†^P < 0.05 vs. EUH-HA, see Table 2. ^‡^P < 0.05 vs. EUH trial. T_re_, Rectal temperature; T_sk_, Skin temperature; BV, Blood volume; Q̇, Cardiac output; HR, Heart rate; SV, Stroke volume; EDV, End-diastolic volume; ESV, End-systolic volume; MAP, Mean arterial pressure; SVR, Systemic vascular resistance.

### Effect of Heat Acclimation on Responses During Upright Exercise

During the 60 min of upright cycling from 20 to 80 min and 100 to 160 min that occurred prior to the bouts of semi-recumbent cycling from 80 to 100 min and 160 to 180 min (Figure 1), HA adaptations were evident following both HA interventions. In the post-HA EUH trials, T_re_ was 0.2 ± 0.2°C and HR was 6 ± 8 beats min^–1^ lower at the end of upright exercise compared to pre-HA EUH trials (*P* = 0.042 and 0.045, respectively). T_re_ and HR responses were similar pre- and post-HA in DEH trials at the end of upright exercise (*P* = 0.297 and 0.184, respectively). Average T_sk_ throughout upright exercise was similar in the pre- and post-HA experimental trials (*P* = 0.348). Ratings of perceived exertion throughout upright exercise decreased from 13 ± 1 to 12 ± 2 in the EUH trials and from 14 ± 1 to 13 ± 1 Borg units in the DEH trials following both HA interventions (*P* = 0.025). Average thermal comfort during upright exercise was not altered by either HA intervention (*P* = 0.170).

### Effect of Heat Acclimation on Responses During Semi-Recumbent Exercise

**FIGURE 2:**
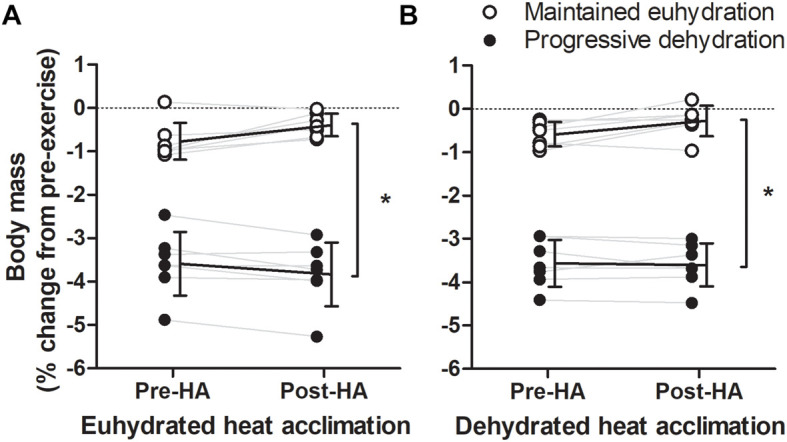
Individual changes in pre-exercising body mass after 180 min of exercise-heat stress pre- and post- euhydrated **(A)** and dehydrated **(B)** heat acclimation (HA). Solid lines are mean and SD of each trial for *n* = 7. *Significant difference from maintained euhydration trial, *P* < 0.001.

Similar to resting responses, HA did not result in an increased BV during exercise (*P* = 0.409, Tables 2, 3). Fluid intake increased following HA by 0.4 ± 0.2 L (from 2.8 ± 0.5 L) and 0.1 ± 0.1 L (from 0.7 ± 0.1 L) in EUH and DEH experimental trials, respectively (*P* = 0.006). This resulted in similar changes in body mass within each trial following exercise pre- and post-HA (*P* = 0.641). Fluid intake in the EUH trials resulted in a stable body mass (i.e., -0.5 ± 0.4% at 180 min) and BV throughout exercise (*P* = 1.00). In contrast, fluid restriction resulted in progressive decreases in body mass of 1.8 ± 0.3% at 100 min and 3.6 ± 0.6% at 180 min (*P* < 0.001, Figure 2). The reduction in body mass with DEH was associated with a 5.9 ± 2.3% (346 ± 197 mL) decrease in BV (*P* = 0.003) that was similar in magnitude between the pre- and post-HA DEH trials (*P* = 0.796). T_re_ during bouts of semi-recumbent exercise were not altered by HA (*P* = 0.62, Tables 2, 3). T_sk_ during semi-recumbent cycling was also unaffected by HA (*P* = 0.640).

**FIGURE 3:**
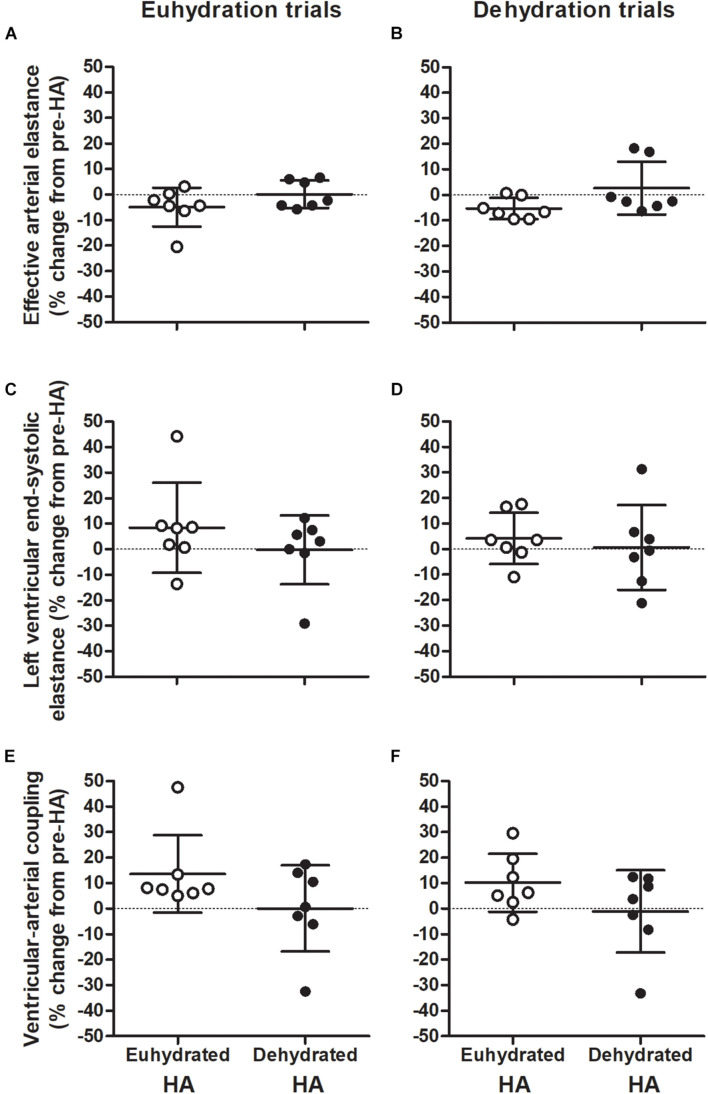
Individual changes in exercising cardiac output **(A,B)**, mean arterial pressure (MAP; **C,D**), and systemic vascular resistance (SVR; **E,F**) during repeated bouts of semi-recumbent cycling in moderate heat with maintained euhydration (left panels) or progressive dehydration (right panels) matched to pre-heat acclimation (HA) levels. Values are relative to responses observed before both euhydrated (open circles) and dehydrated exercise HA (closed circles) interventions. Data are mean ± SD for *n* = 7. Cardiac output: Main effect of hydration (*P* = 0.001). SVR: HA intervention by state interaction (*P* = 0.018). *Significant difference from pre-HA, *P* < 0.05.

**FIGURE 4:**
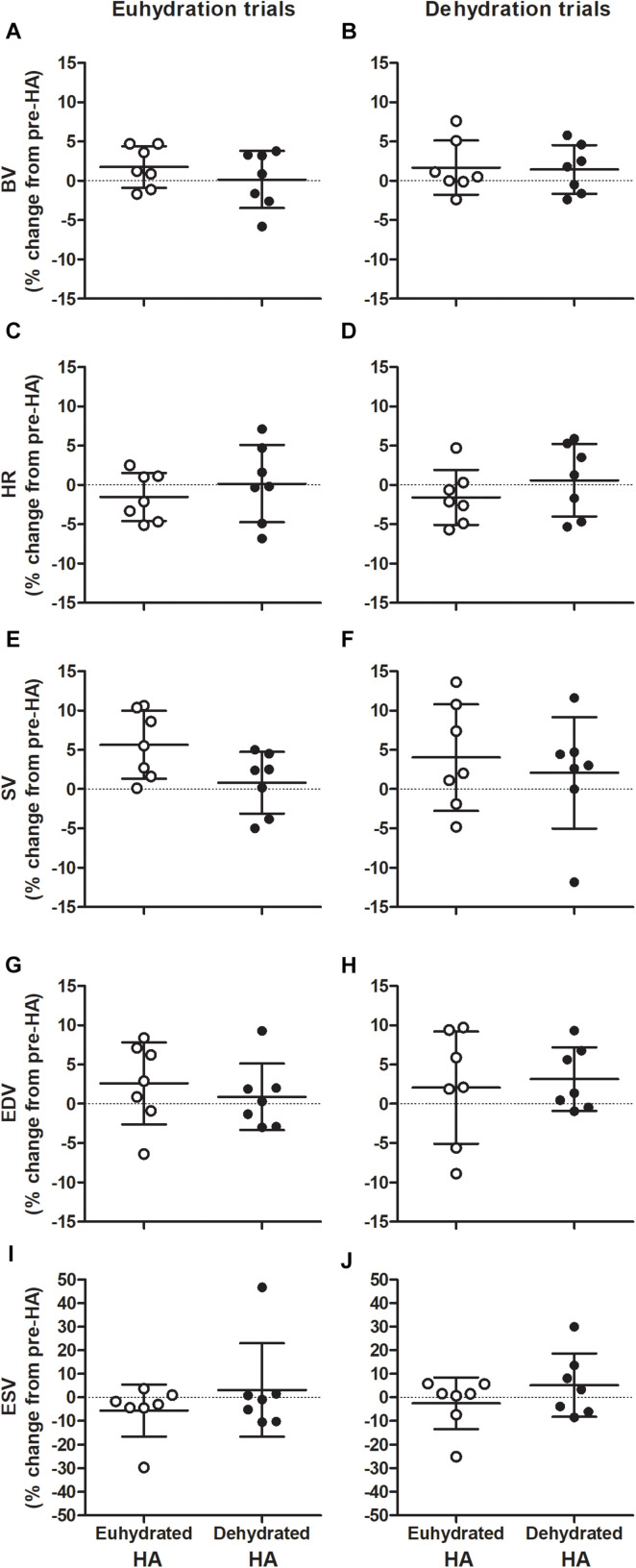
Individual changes in exercising blood volume (BV; **A,B**), heart rate (HR; **C,D**), stroke volume (SV; **E,F**), end-diastolic (EDV; **G,H**), and end-systolic volumes (ESV; **I,J**) during repeated bouts of semi-recumbent cycling in moderate heat with maintained euhydration (left panels) or progressive dehydration (right panels) matched to pre-heat acclimation (HA) levels. Values are relative to responses observed before both euhydrated (open circles) and dehydrated exercise HA (closed circles) interventions. Data are mean ± SD for *n* = 7. BV: Main effect of hydration (*P* = 0.009). HR: Main effect of hydration: (*P* = 0.001). SV: Main effect of HA state (*P* = 0.045) and hydration (*P* = 0.001). EDV: Main effect of hydration (*P* = 0.001).

**FIGURE 5:**
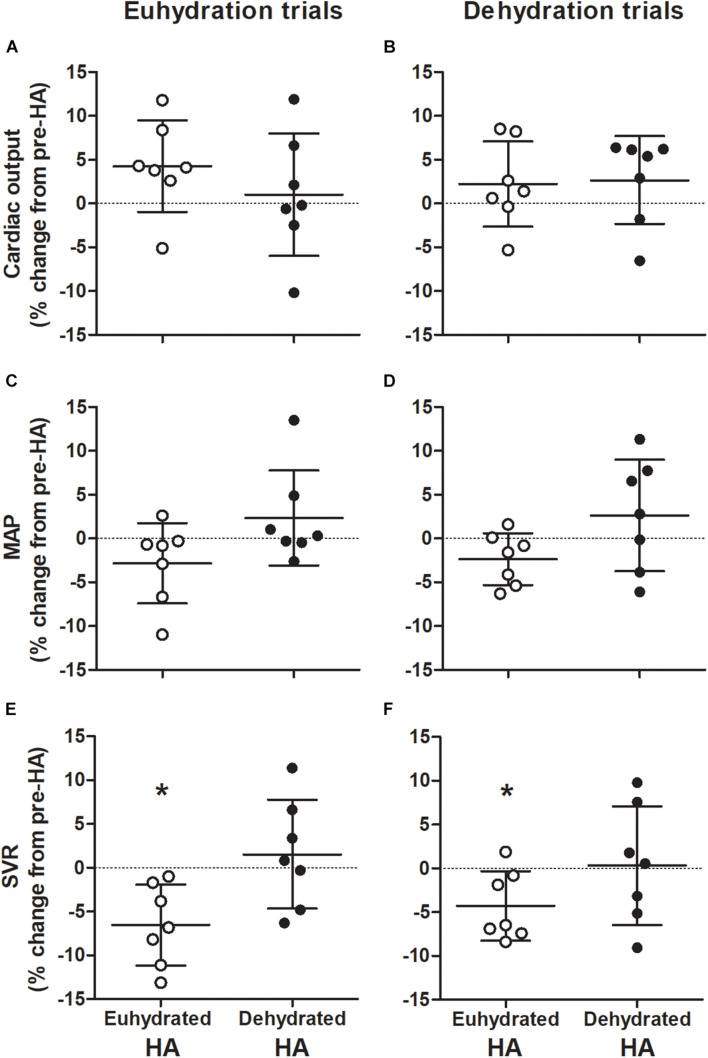
Individual changes in effective arterial elastance **(A,B)**, left ventricular end-systolic elastance **(C,D)**, and ventricular-arterial (VA) coupling **(E,F)** during repeated bouts of semi-recumbent cycling in moderate heat with maintained euhydration (left panels) or progressive dehydration (right panels) matched to pre-heat acclimation (HA) levels. Values are relative to responses observed before both euhydrated (open circles) and dehydrated exercise HA (closed circles) interventions. Data are mean ± SD for *n* = 7. Effective arterial elastance: Main effect of hydration (*P* < 0.001). Left ventricular end-systolic elastance: HA intervention by state interaction (*P* = 0.040). VA coupling: Main effect of HA intervention (*P* = 0.044).

There was no effect of HA on Q̇ throughout semi-recumbent cycling (*P* = 0.129, Figure 3 and Tables 2, 3). This was associated with a similar exercising HR (*P* = 0.500) and unchanged diastolic or systolic LV volumes following HA (*P* = 0.133 and 0.841, respectively, Figure 4). Exercising MAP was also similar to pre-HA values following both HA interventions (*P* = 0.738). SVR tended to be altered by HA (*P* = 0.062) and there was a significant hydration strategy and HA status interaction effect (*P* = 0.018, Figure 3 and Tables 2, 3). Exercising effective arterial elastance (*P* = 0.171), end-systolic elastance (*P* = 0.113) and ventricular-arterial coupling (*P* = 0.071) were similar between the pre- and post-HA trials (Figure 5).

### Exercise Induced Dehydration Following Acclimation

Thermal, hematological, LV volumes, and central hemodynamic responses throughout DEH exercise were largely unchanged by HA state or strategy (Tables 2, 3). Relative to EUH, exercise with DEH was associated with a 0.5 ± 0.3°C greater increase in T_re_ (*P* = 0.017) and similar T_sk_ throughout exercise post-HA (*P* = 0.624, Tables 2, 3). Q̇ declined during exercise in all DEH trials (*P* < 0.001) and was 2.0 ± 1.1 L min^–1^ lower than EUH at 180 min following HA interventions (*P* = 0.001). The fall in Q̇ with DEH was associated with a lower SV (24 ± 9 mL, *P* < 0.001) and higher HR (14 ± 9 beats min^–1^, *P* = 0.015) at 180 min compared to EUH during post-HA trials. The lower SV with DEH was related to a 26 ± 14 mL lower EDV at 180 min post-HA following both interventions (*P* = 0.008), as ESV was maintained throughout exercise (*P* = 0.87, Tables 2, 3). There was a main effect of exercise time on MAP (*P* = 0.006), however, responses were similar between hydration conditions (*P* = 0.715) with no interaction effects observed (*P* = 0.858). No main effect of hydration on SVR was observed (*P* = 0.366, Tables 2, 3). Effective arterial elastance increased throughout all DEH trials (*P* < 0.001) and was 24 ± 16% higher than EUH at 180 min following both interventions (*P* < 0.001). LV end-systolic elastance was similar between EUH and DEH trials post-HA (*P* = 1.00) while ventricular arterial coupling decreased slightly during DEH exercise in post-HA trials (both *P* = 0.006) but did not differ from EUH at 180 min (*P* = 0.131).

## Discussion

In the present study, trained individuals underwent two controlled-HR HA interventions with different fluid intake strategies. Daily pre-exercise euhydrated body mass was maintained, or moderate (∼2.9%) dehydration was achieved during each 10-day HA period *via* prescribed fluid intake. Neither HA intervention significantly altered PV or red cell volume, and thus total BV was similar between all experimental trials. Furthermore, fluid intake strategy during HA did not influence thermal, cardiac and systemic hemodynamic responses at rest and during bouts of semi-recumbent cycling under moderate heat stress with maintained euhydration or dehydration of ∼3.6% body mass, as responses were similar to those observed pre-HA (Figure 3).

### Hydration Strategy During Heat Acclimation

Adaptations observed throughout HA were largely similar between hydration interventions, as were the responses to EUH exercise during the post-HA trials. HA with daily dehydration has previously demonstrated greater increases in post-exercise aldosterone and a tendency for greater PV expansion compared to euhydrated HA during a 5-day cross-over intervention (Garrett et al., 2014). Daily dehydration (∼1.8–2.6%) during HA has also been shown to increase and maintain PV and BV over 28 days (Patterson et al., 2004b, 2014), however, these responses were not compared to a euhydrated control intervention. The findings of the present study more closely reflect those of others (Neal et al., 2016a, b; Pethick et al., 2019), in that matched levels of daily whole-body fluid loss in the order of ∼2.5% during exercise in the heat did not influence thermal, hematological, cardiovascular or systemic hemodynamic adaptations with exercise HA. The reason PV and BV were not altered by either HA intervention in the present study is unclear. Each HA intervention provided significant thermal strain, with a similar daily T_re_ of ∼38.4°C maintained throughout each exposure *via* exercise at a controlled HR. It is possible that the moderate levels of daily dehydration (i.e., ∼3% body mass) did not provide a sufficient stimulus for renal water conservation (Kenefick et al., 2007), and that more pronounced dehydration (i.e., 4–5% of body mass) might enhance sodium reabsorption independently of the levels of whole-body heat stress achieved during HA in the present study (van den Heuvel et al., 2020). Furthermore, similar to controlled hyperthermia HA, where the workload to maintain a T_re_ of 38.5°C in a given environment may be lower when fluid intake is restricted (Garrett et al., 2014), exercising HR was maintained by substantially larger reductions in workload during HA with dehydration (∼30 W or 17% of initial workload, compared to ∼17 W or 9% for euhydrated HA). It is therefore possible that the incurred deficit in body water did not result in an additive stimulus for vascular adaptations to HA compared to exercising with maintained euhydration. For example, a decline in Q̇ and V̇O_2_ occurs as workload decreases to maintain exercising HR in the heat (Wingo and Cureton, 2006). With dehydration during HA, it is plausible that V̇O_2_ and Q̇ were ∼150 mL min^–1^ and ∼1 L min^–1^ lower, respectively, throughout HA with dehydration compared to maintained euhydration, assuming proportionate reductions relative to the lower workload achieved. Permissive dehydration during exercise HA at a constant workload would potentially elicit far greater thermal, hematological, cardiovascular, and fluid regulatory strain. This may occur *via* greater whole-body temperatures and sweat losses, and increased sodium re-absorption and reductions in renal blood flow and MAP during constant load exercise compared to altering intensity to maintain a T_re_ ∼38.5°C (Smith et al., 1952; Houmard et al., 1990; González-Alonso et al., 1998; Melin et al., 2001; Otani et al., 2013). The effects of such an intervention on hematological and cardiovascular adaptations to HA is warranted. It is also possible that participants exhibited some basal levels of natural acclimatization as residents of a hot climate. To control for this factor, outdoor exercise was limited for a 3-week period prior to commencing each intervention and a washout period of ∼10 weeks was observed between HA interventions. Furthermore, measurements of resting absolute BV and PV were similar between all pre-HA trials and remained unchanged with HA (Tables 2, 3 and Figure 4). As such, it appears that neither intervention influenced PV or BV. Future studies may further explore the possible contributions of hydration strategy during HA on hematological adaptations, perhaps *via* beginning HA exposures in a hypohydrated state or withholding food and fluid intake for a given period following each exposure to elicit more pronounced levels of whole-body dehydration and stimulate fluid regulatory processes.

A decrease in exercising HR is another key adaptation proposed to permit an enhanced diastolic filling pressure (Senay, 1986) and increase SV following HA (Rowell et al., 1967; Wyndham et al., 1968; Nielsen et al., 1993). However, in contrast to our hypothesis that dehydration during HA would lead to greater LV filling, EDV and SV were not notably altered by either HA intervention (both ∼4 mL). As noted above, these responses were associated with similar BV and HR at rest and during exercise between the pre- and post-HA trials (Figure 3 and Tables 2, 3). Whether the alterations in SV and Q̇ that occur with HA in some studies are due to changes in BV, HR, and/or other factors remain unclear. Observations from acute manipulations of BV and HR indicate that several integrated peripheral and central cardiovascular adjustments may instead occur with HA to regulate peripheral and systemic oxygen delivery, *via* alterations in flow. In support of this, infusion of ∼600 mL packed cells does not alter Q̇, SV, leg blood flow, or V̇O_2_ at rest and during single-leg knee extensor exercise (González-Alonso et al., 2006). Furthermore, reduced HR *via* β-adrenoreceptor blockade during exercise augments SV without altering Q̇ or forearm and cutaneous blood flow (Fritzsche et al., 1999; Trinity et al., 2010). Within the HA literature, adaptive responses related to cardiovascular function are also varied. For example, exercising Q̇ has been reported to decrease (Wyndham, 1951), remain unchanged (Rowell et al., 1967; Wyndham et al., 1968), or increase (Nielsen et al., 1993) following HA, while data regarding human cardiac volumes, function and systemic blood flow is limited. Recently, passive and active HA regimens were shown to minimally enhance left atrial and ventricular diastolic volumes at rest; however, these responses were not correlated with an increase in BV or lowered HR (Parsons et al., 2020; Wilson et al., 2020). Passive HA with controlled hyperthermia has also failed to induce changes in resting HR, EDV, and SV in thermoneutral conditions (Trachsel et al., 2020). Furthermore, during passive heating, HR, SV, LV systolic function, and Q̇ were unaltered by HA despite an attenuated decrease in EDV (Trachsel et al., 2020). The present study extends these findings and indicates that diastolic filling, SV, and Q̇ are not enhanced during exercise following controlled-HR exercise-HA with varying fluid intake strategies, where BV and HR remain unchanged.

### Acute Exercise-Induced Dehydration Following Heat Acclimation

Daily exercise-induced dehydration (∼2.9% body mass loss) did not attenuate the deleterious effects of acute mild and moderate dehydration on cardiovascular function during submaximal semi-recumbent exercise. Previous studies have reported an attenuated increase in body temperature and HR during exercise-induced dehydration following HA with fluid restriction (Patterson et al., 2004a, 2014). Together with a greater BV following HA with dehydration, a blunted impairment in LV diastolic filling during acute exercise-induced dehydration was expected. In contrast to this hypothesis, however, when fluid intake was restricted so that matched (∼1.8 and 3.6% body mass loss) levels of exercise-dehydration occurred, similar impairments in thermoregulatory and cardiovascular function were observed pre- and post-HA. Similarly to our previous observations following euhydrated HA (Travers et al., 2020a), during the DEH trials Q̇ was ∼2 L min^–1^ lower than the EUH trials at 180 min, and associated with similar elevations in body temperature and HR, and declines in BV, LV diastolic filling, and SV to the pre-HA DEH trials (Tables 2, 3 and Figures 3, 4). The increased HR throughout the DEH trials was likely due to the elevated T_re_ and increased sympathoadrenal activity (Gonzalez-Alonso et al., 1995; González-Alonso et al., 1998), accompanying reductions in BV. Lowering HR *via* β-adrenergic blockade restores SV during exercise in the heat (Trinity et al., 2010), presumably due to a greater duration of diastolic filling. Furthermore, dextran infusion to maintain BV during exercise with fluid restriction attenuates the decline in SV and the increase in HR such that Q̇ is maintained (Montain and Coyle, 1992). Taken together, these findings indicate that elevations in HR and reductions in BV with acute dehydration independently contribute to reductions in preload of the LV during exercise in the heat (Watanabe et al., 2020), and that these thermal and cardiovascular perturbations persist following HA with varying fluid intake strategies.

### Limitations and Methodological Considerations

The T_re_, HR, and SV responses were largely similar during bouts of semi-recumbent exercise pre- to post-HA and therefore the level of HA adaptation appears small. However, the similarities in responses may be related to the relatively brief bouts of submaximal exercise in the semi-recumbent position, which promotes venous return and LV preload, under moderate hyperthermia. In contrast, over the 10-day HA interventions, HR was lowered during upright fixed workload exercise and workload increased during controlled heart rate exercise. Furthermore, during the experimental EUH trials following both HA interventions, T_re_ and HR were slightly lower during the periods of upright exercise. Finally, while the present study consists of a small sample of male participants, the cross-over design adds to the limited data within the literature exploring intra-individual responses to different HA regimens. Notwithstanding, additional studies are needed to explore the responses of more pronounced levels of dehydration and whole-body hyperthermia in both men and women.

## Conclusion

The findings of the present study indicate that hydration strategy during exercise-HA with controlled HR in trained individuals did not influence the PV or BV adaptive response. Additionally, during EUH semi-recumbent exercise under moderate heat stress, LV volumes, Q̇, and systemic hemodynamics were not notably altered by HA, with similar responses between interventions. Furthermore, matched levels of progressive DEH to ∼3.6% body mass loss before and after HA were associated with strikingly similar elevations in T_re_ and HR and lowered BV, SV, and Q̇ during exercise. As such, further research is required to explore the role of increased BV on cardiovascular responses during exercise following HA.

## Data Availability Statement

The original contributions presented in the study are included in the article/supplementary material, further inquiries can be directed to the corresponding author.

## Ethics Statement

The studies involving human participants were reviewed and approved by Anti-Doping Lab Qatar Research Ethics Committee (Approval number F201500105). The patients/participants provided their written informed consent to participate in this study.

## Author Contributions

GT, JP, and JG-A designed the study. GT, NR, AS, and DN collected the data. All authors interpreted and analyzed the data, contributed to drafting the work, revising it critically for important intellectual content, and approved the final manuscript.

## Conflict of Interest

The authors declare that the research was conducted in the absence of any commercial or financial relationships that could be construed as a potential conflict of interest.

## Publisher’s Note

All claims expressed in this article are solely those of the authors and do not necessarily represent those of their affiliated organizations, or those of the publisher, the editors and the reviewers. Any product that may be evaluated in this article, or claim that may be made by its manufacturer, is not guaranteed or endorsed by the publisher.
